# Rhoifolin regulates oxidative stress and proinflammatory cytokine levels in Freund’s adjuvant-induced rheumatoid arthritis via inhibition of NF-κB

**DOI:** 10.1590/1414-431X20209489

**Published:** 2020-05-08

**Authors:** Shanqin Peng, Congqi Hu, Xi Liu, Lei Lei, Guodong He, Chenming Xiong, Wenqian Wu

**Affiliations:** 1 Guangzhou University of Chinese Medicine, Guangzhou, China; 2 Department of Gastroenterology, The 455th Hospital of Chinese People’s Liberation Army, Shanghai, China; 3 YouJiang Medical University for Nationalities, Baise, China; 4 Department of Hepatobiliary Surgery, The First Affiliated Hospital of Guangzhou University of Traditional Chinese Medicine, Guangzhou, China; 5 Department of Traditional Chinese Medical Gynecology, Wenzhou Hospital of Chinese Medicine, Wenzhou, China

**Keywords:** Flavonoids, Rheumatoid arthritis, Oxidative stress, Anti-inflammatory, Cytokines

## Abstract

Rheumatoid arthritis (RA) is an autoimmune disease of knee joints involving pain and inflammation. Rhoifolin is a plant flavonoid known to have antioxidant and anti-inflammatory properties. This study was taken to identify the effect of rhoifolin on complete Freund’s adjuvant (CFA)-induced arthritis in the rat model. Treatment with rhoifolin (10 and 20 mg/kg) showed a significant improvement in the overall health parameters such as paw edema and weight loss. This improvement in morphological parameters corroborated the findings with gross morphological changes observed in the histopathological analysis. Rhoifolin treatment also caused a significant decrease in oxidative stress, evident from changes in intracellular levels of glutathione, glutathione peroxidase, malondialdehyde, and superoxide dismutase in the articular cartilage tissue. Moreover, proinflammatory cytokines, tumor necrosis factor (TNF)-α, interleukin(IL)-1β, and IL-6 showed a significant downregulation of gene expression and intracellular protein concentration levels. The NF-κB pathway showed a significant attenuation as evident in the significant reduction in the levels of NF-κB p65 and p-IκB-α. These results indicated that rhoifolin can be a natural therapeutic alternative to the extant regimens, which include non-steroidal anti-inflammatory drugs and immunosuppressants. Additionally, the antioxidant and anti-inflammatory action of rhoifolin was probably mediated by the NF-κB pathway. However, the exact target molecules of this pathway need to be determined in further studies.

## Introduction

Arthritis is a condition that mostly affects elderly individuals and includes symptoms such as stiffness of joints, pain, and dysfunction (1,2). Rheumatoid arthritis (RA) is an autoimmune disorder that is characterized by chronic, systemic, and progressive inflammation of the synovial membranes. This inflammation of synovial membranes results in the degradation of articular cartilages and the associated subchondral bones (3). Despite extensive studies, the exact cause of RA remains elusive (3). However, recent studies have shown that free radical generation resulting in increased oxidative stress may be an important mechanism behind RA progression (4,5). Elevated levels of several proinflammatory cytokines such as interleukin-6 (IL-6), IL-1β, and tumor necrosis factor-α (TNF-α) have been shown to play a key role in the progression of inflammation in RA (6–9). Additionally, it has been suggested that it is TNF-α that drives IL-1 production (10). Therefore TNF-α and IL-1 have been extensively used as markers for the detection of progression of RA (6,7 [Bibr B08]). The transcription factor NF-κB is a key regulator of the inflammation associated with RA (11). TNF-α and IL-1 are both inducers and targets of NF-κB. Therefore NF-κB helps in the amplification of the pro-inflammatory signal. Additionally, NF-κB is known to be an inducer of the expression of over 150 genes including chemokines, cytokines, growth factors, and cell adhesion mediators (11). Several studies have shown the efficacy of NF-κB inhibitors as a potential approach for the management of RA (11,12).

The extant therapeutic approaches for the management of RA target different pathways involved in its progression. These therapies include the use of non-steroidal anti-inflammatory drugs (NSAIDs), immunosuppressants (13,14), disease-modifying anti-rheumatic drugs (15), biological agents (16), and corticosteroids (17). These therapies have variable effectiveness and several side effects, namely gastrointestinal upset, peptic ulcers, and toxicities at non-systemic and systemic levels (18–20). To avoid these complications, the use of natural therapeutic agents has gained considerable interest in recent years (21).

Several plant-derived products are known for their anti-arthritic properties (22). These bio-active products possess anti-oxidant and anti-inflammatory properties and therefore can provide relief from pain by preventing the generation of free radicals (23). Rhoifolin is a flavanone first extracted from *Rhus succedanea* (24). Rhoifolin has been shown to possess anti-inflammatory, antioxidant (25), and anticancer (26) properties. However, to our knowledge, rhoifolin has never been tested for its anti-arthritic properties. Therefore, this study was designed to test the anti-inflammatory properties of rhoifolin in the rat RA model induced by Freund’s adjuvant.

## Material and Methods

Wistar rats (weighing 145 to 155 g) were provided by the animal house of the Guangzhou University of Chinese Medicine. The animals were kept under a 12-h light/dark circadian cycle and under controlled conditions of temperature and humidity. The animals were fed a standard rat diet and had water *ad libitum*. All the animals’ upkeeping and experimental procedures were carried out following the ethical standards of the Declaration of Helsinki. The animal experiments were conducted with prior approval from the institutional review board of Guangzhou University of Chinese Medicine (approval number: TCMF1-2018033). All efforts were made to minimize the number of animals and their suffering.

### Acute toxicity and identification of optimal dosage

Studies on the acute toxicity of rhoifolin (Sigma Aldrich, USA) were performed on control animals. Graded rhoifolin doses were dissolved in dimethyl sulfoxide (DMSO,1%, v/v) (Sigma Aldrich, USA). The doses were administered orally at concentrations from 10 to 50 mg/kg and the animals were kept on a 10-day observation period. Health parameters such as diet, changes in weight, fluid intake, and psychomotor changes were measured. Rhoifolin did not show any toxicity at all the tested doses. Therefore, we selected the lowest two doses i.e., 10 and 20 mg/kg for further experiments.

### Establishment of arthritis model

Arthritis was induced in rats by injecting 0.6 mg complete Freund’s adjuvant (CFA) (Sigma Aldrich) containing 10 mg/mL of heat-killed *Mycobacterium tuberculosis* subcutaneously at the base of the tail. The animals were assigned to six experimental groups at random with six animals per group: 1) healthy group, no induction, no rhoifolin; 2) control group, animals that received PBS+1% DMSO; 3) CFA group; 4) CFA+10 mg/kg rhoifolin group; 5) CFA+20 mg/kg rhoifolin group; 6) CFA+10 mg/kg indomethacin group.

Rhoifolin was dissolved in 1% DMSO and administered orally by gavage in 3 mL volume doses daily. Rhoifolin treatment began 24 h after the induction of arthritis by CFA and continued for 1 month with one dose each day. The diameter of the right paw joint and body weight were measured every five days.

### Estimation of hepatic and kidney toxicity parameters

On the completion of the experiment, blood was drawn via retro-orbital plexus. Blood samples were centrifuged at 1300 *g* for 30 min at 4°C for separation of serum. Hepatic toxicity of rhoifolin was assessed by estimating aspartate aminotransferase (AST) and alanine aminotransferase (ALT) levels in blood serum using kits (CRESCENT Diagnostics, KSA). Kidney toxicity of rhoifolin was determined by estimating blood urea nitrogen and creatinine levels, using biochemical kits (ACCUREX, Biomedical Pvt. Ltd, India). The animals were euthanized at the end of the experiment with 500 mg of ketamine (*im*). The arthrotomy for harvesting femoral condyles was conducted and the cartilage tissue samples were taken from the distal femur medial condyles.

### Estimation of hematological parameters

Hematological parameters were measured at the end of the study. Blood was drawn by retro-orbital puncture from all groups and was stored in anticoagulant and centrifuged at 3000 *g* for 30 min at 4°C to collect the serum. Standard rat blood hematology reagents were used to determine red and white blood cell counts, hemoglobin, and erythrocyte sedimentation rate.

### Antioxidant marker estimation

Articular tissue from sacrificed rats was extracted. An equal weight of tissue was homogenized in PBS (10% w/v) and centrifuged at 13000 *g* for 1 h at 4°C. Assay of supernatants was performed for estimating the concentration of glutathione (GSH) using a glutathione GSH/GSSG assay kit (Sigma Aldrich), glutathione peroxidase (GPx) using a glutathione assay kit (Cayman Chemicals, USA), malondialdehyde (MDA) using a lipid peroxidation (MDA) assay kit (Abcam, USA), and superoxide dismutase (SOD) using a superoxide anion assay kit (Sigma Aldrich). All the experimental procedures were carried out following the respective manufacturer’s protocols.

### Estimation of cytokine levels

The blood sera were obtained as mentioned above. The levels of TNF-α, IL-1β, and IL-6 in the sera of CFA-induced animals were determined using an ELISA kit (Sigma Bioscience, USA), according to the manufacturer’s instructions.

Total blood RNA was extracted using the RiboPure™ Blood RNA Isolation kit (Thermo Fisher Scientific, USA). Geneious software (USA) was used for designing primers for qRT-PCR. The following primers were used for qRT-PCR: IL-6 (5′-CATTCTGTCTCGAGCCCACC-3′, 5′-GCAACTGGCTGGAAGTCTCT-3′); TNF-α, (5′-CTGAAGTCTGCGTCTGTCGT-3′, 5′-GTTCCACAGGGGTCTTGGAG-3′); IL-1β (5′-CCTCTGCCTCTTGACGATGG-3′, 5′-AGGACGTGCGGCAAGTATAG-3′). GAPDH (5′-GTGCCAGCCTCGTCTCATAG-3′, 5′-AGAGAAGGCAGCCCTGGTAA-3′) was used as an internal control. Three technical replicates for each biological replicate were used. RNA was quantified using Qubit fluorometer (Thermo Fisher).

The following components were added tothe PCR master-mix: 1.5 μL cDNA, 1 μL (5 pm/μL) each primer, and 5 μL DyNAmo Flash SYBR Green (Thermo Fisher) (2×). The PCR was cycled 42 times with the following conditions: 10 s at 95°C, 40 cycles for 22 s at 95°C, 60 s at 60°C. ABI Prism 7500 (Applied Biosystems, USA) was used for the qRT-PCR run. The Ct (threshold cycle) value was normalized and quantified using the Ct value of GADPH. The 2^−ΔΔCt^ method (27) was used to calculate the relative expression.

### Western blot analysis

Antibodies for NF-κB p65, p-NF-κB p65 were procured from Santa Cruz Biotechnology, Inc. (USA). The anti-IκB-α and anti-p-IκB-α antibodies were procured from Cell Signaling Technology, Inc. (USA). The total protein in the synovial cavity was extracted and estimated using Bradford’s method. Total protein (10 μg) from bronchoalveolar fluid was loaded in each well in triplicate. Electrophoretic transfer of proteins was performed on PVDF membranes. After incubation for two hours and washing with Tris-buffered saline containing 0.2% Tween 20 (TBST) and blocking with 4% non-fat dry-milk, the membranes were incubated with primary antibodies overnight. Finally, the membranes were incubated with HRP. ECL detection system (Thermo Fisher) was used to develop the membranes. The relative band intensity was determined using ImageJ software (NIH, USA).

### Histopathological analysis of rat knee

Knee cartilage tissues were isolated and fixed in a 12% neutral buffered formaldehyde solution for 72 h at 4°C. Decalcification of tissues was performed in formic acid for 6 days. Subsequently, the tissues were dehydrated in increasing concentrations of ethanol. The dehydrated tissues were cleared with xylene and fixed in paraffin. Sections of five to seven microns were cut using a microtome and were stained with hematoxylin and eosin with safranin O/Fast green. The morphological differences between the sections of different treatment groups were scored following the Mankin scoring system (Supplementary Table S1). The scoring was performed twice by two independent researchers who were blinded to the treatment experiment.

### Statistical analysis

All data are reported as means±SE. ANOVA was used to compare the results with Newmann-Keuls multiple comparison test. Statistical analysis was performed with GraphPad Prism software (USA) with a P value ≤0.05 indicating a significant difference.

## Results

### Hepatic and kidney toxicity

Rhoifolin administration showed no significant difference in serum AST and ALT levels compared to the control animals. Similarly, the levels of blood urea nitrogen and creatinine showed no significant difference between control and rhoifolin-treated animals (Supplementary Table S2). These results indicated that rhoifolin did not have hepatic or kidney toxicity effects in this study.

### Rhoifolin attenuated CFA-induced edema

**Figure 1:**
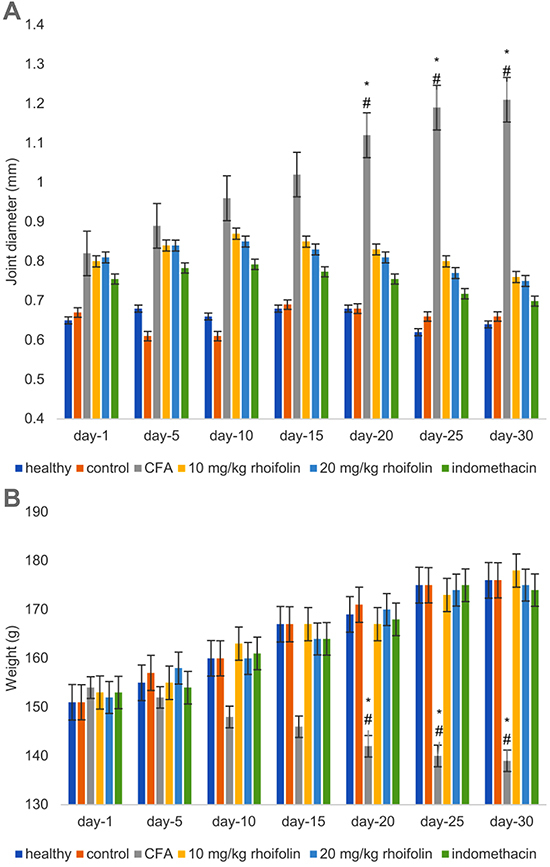
Effect of rhoifolin treatment on paw edema as estimated by measuring the joint diameter on complete Freund’s adjuvant (CFA)-induced edema (**A**). Effect of rhoifolin treatment on the body weight of CFA-induced animals (**B**). Data are reported as means±SE. ^#^P≤0.05 compared to 10 mg/kg rhoifolin group. *P≤0.05 compared to 20 mg/kg rhoifolin group (ANOVA).

Induction of arthritis with CFA caused significant swelling and edema in the rat paws, indicating a successful establishment of the model (Figure 1A). The oral administration of rhoifolin showed a gradual and significant decrease in edema compared to the CFA group starting from the 20th day. This decrease was found to be equivalent in both treatment doses, as the 10- and 20-mg/kg rhoifolin groups showed similar attenuation in edema measured by the joint diameter. Moreover, it was observed that the attenuation of edema in rhoifolin treatment groups was similar to the indomethacin group.

### Rhoifolin prevented weight loss in CFA-induced arthritis

CFA-induced arthritis caused significant weight loss in the experimental animals compared to the healthy group (Figure 1B). However, treatment with rhoifolin resulted in the prevention of weight loss. The weight loss in the two rhoifolin treatment groups had no significant difference compared to the healthy group. Moreover, there was no significant difference in the weight loss between the rhoifolin and the indomethacin-treatment groups, indicating that rhoifolin was as efficient as indomethacin in the prevention of weight loss in CFA-induced arthritis.

### Effect of rhoifolin treatment on hematological parameters

**Figure 2:**
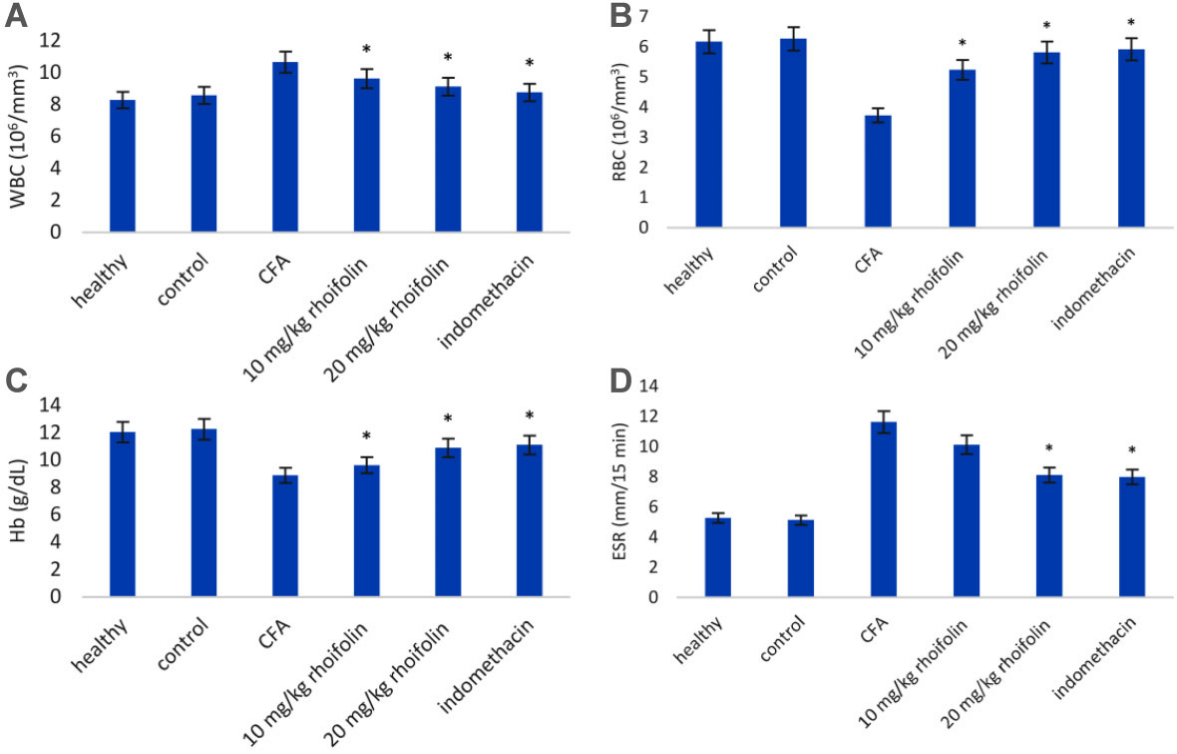
Effect of rhoifolin treatment on hematological parameters in complete Freund’s adjuvant (CFA)-induced arthritis in rats. Data are reported as means±SE. *P≤0.05 compared to CFA-induced group (ANOVA).

The CFA-induced group showed a significant deterioration of hematological parameters compared to the healthy group (Figure 2). Treatment with rhoifolin caused a significant improvement in all four hematological parameters. This result also indicated a general improvement in the overall health of CFA animals with rhoifolin treatment.

### Antioxidant activity of rhoifolin in CFA-induced arthritis

**Figure 3:**
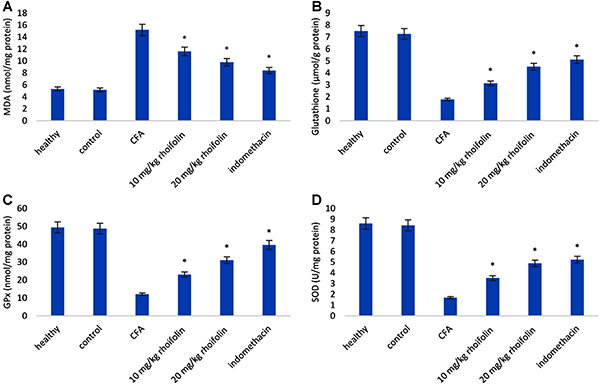
Effect of rhoifolin treatment on antioxidant marker enzymes. Data are reported as means±SE. *P≤0.05 compared to complete Freund’s adjuvant (CFA)-induced group (ANOVA). MDA: malondialdehyde; GPx: glutathione peroxidase; SOD: superoxide dismutase.

Induction of arthritis caused a significant increase in oxidation levels in the articular tissue (Figure 3). The levels of GSH, GPx, and SOD were significantly downregulated and the levels of MDA were significantly upregulated in the arthritic animals compared to the healthy group. Treatment with rhoifolin showed a significant improvement in the oxidation state in the articular tissue compared to the arthritis group animals. Moreover, the highest rhoifolin concentration group (20 mg/kg rhoifolin) showed an oxidation state similar to the indomethacin group.

### Rhoifolin attenuated cytokine levels in CFA-induced arthritis

**Figure 4:**
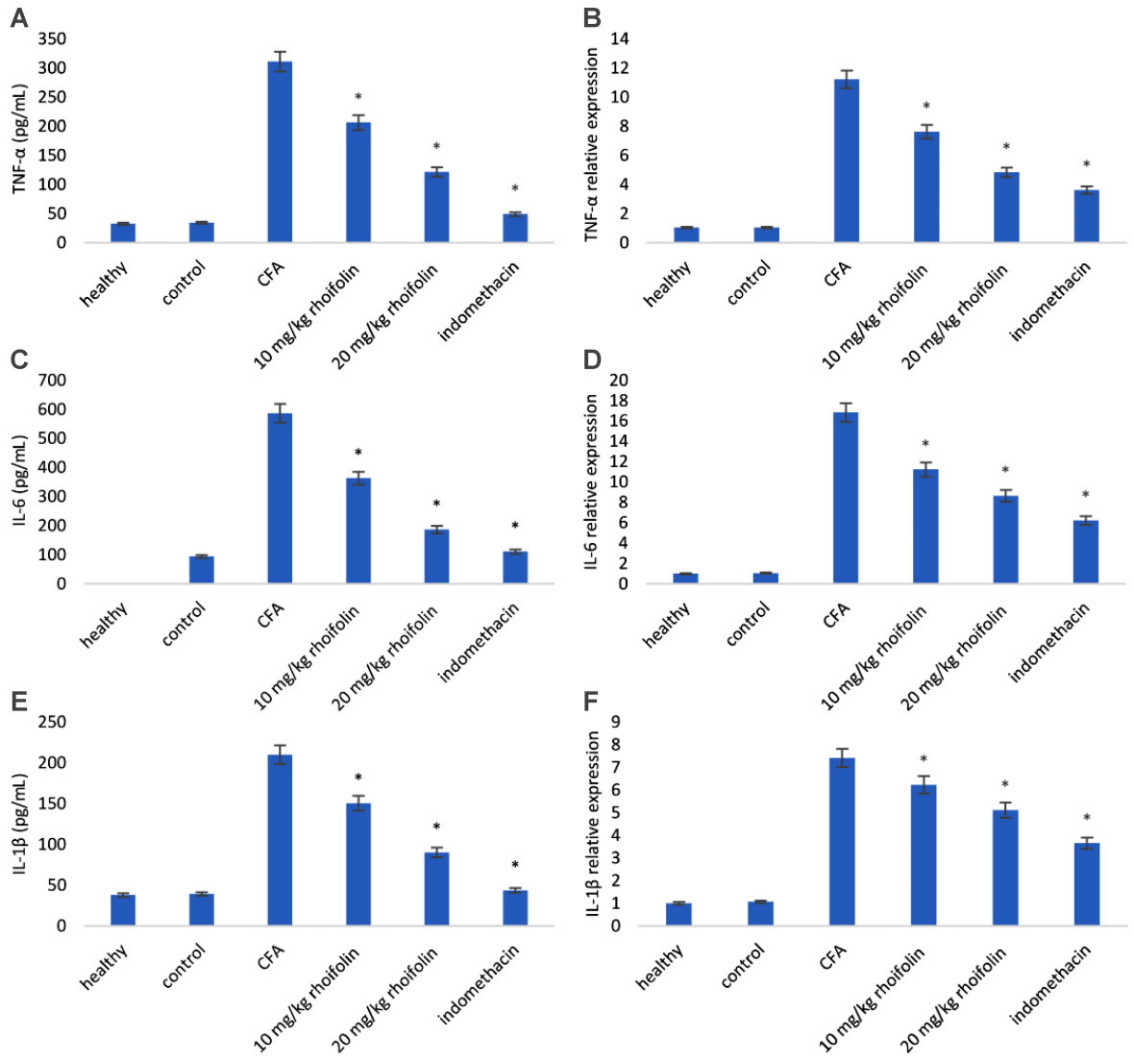
Effect of rhoifolin treatment levels of tumor necrosis factor (TNF)-α and relative expression (**A** and **B**), interleukin (IL)-6 and relative expression (**C** and **D**), and IL-1β and relative expression (**E** and **F**). Data are reported as means±SE. *P≤0.05 compared to complete Freund’s adjuvant (CFA)-induced group (ANOVA)

ELISA and qRT-PCR analysis showed that the induction of arthritis caused a significant increase in the levels of TNF-α, IL-1β, and IL-6 compared to the healthy group (Figure 4). However, rhoifolin treatment caused a significant reduction of these cytokines compared to the CFA-induced arthritis group. It was also observed that cytokine levels in the highest rhoifolin concentration group (20 mg/kg) was lower than the indomethacin group.

### Rhoifolin attenuated NF-&mac_kgr;B levels in CFA-induced arthritis

**Figure 5:**
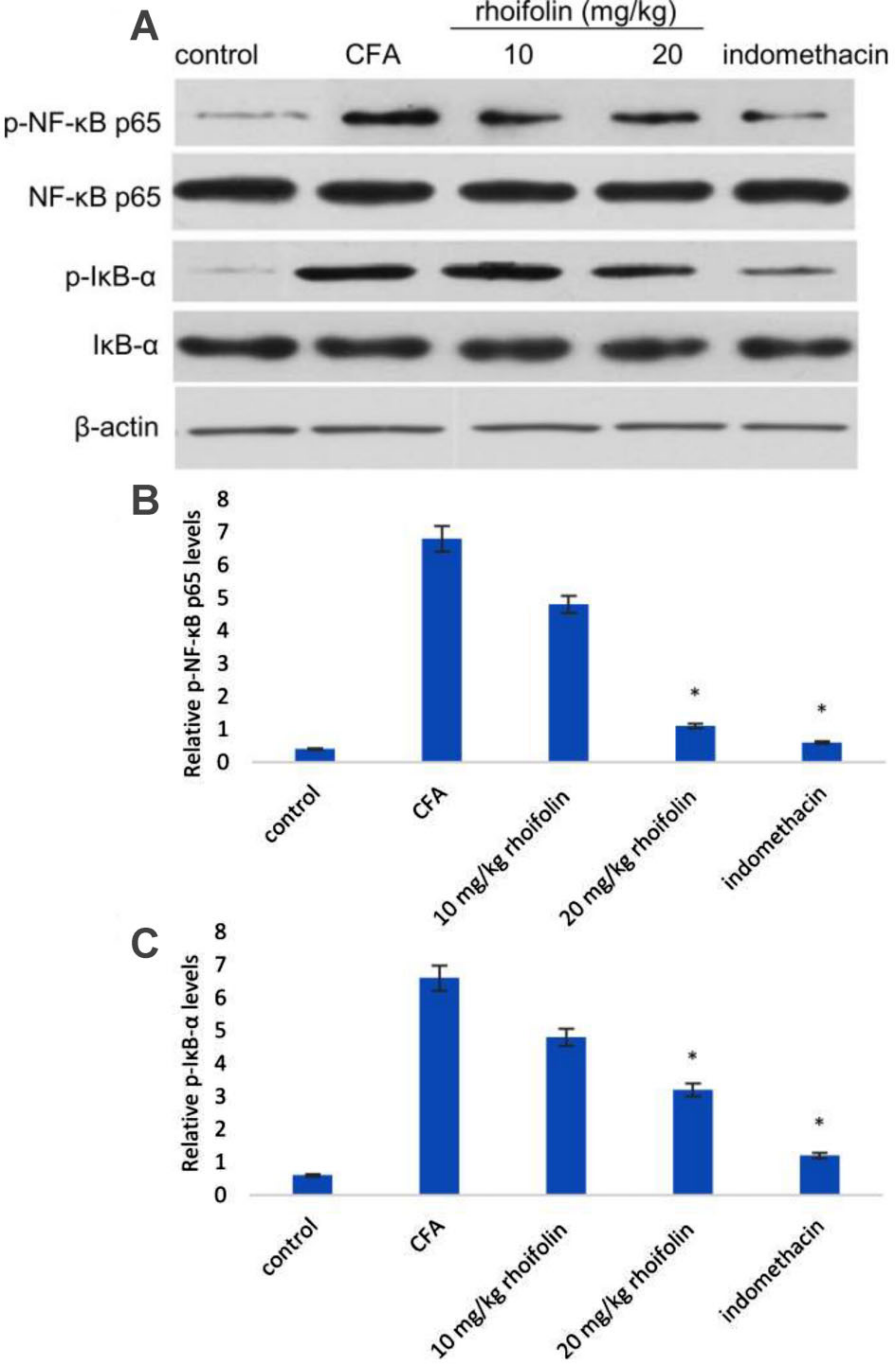
Western blot analysis of levels of phosphorylated and non-phosphorylated forms of NF-κB p65 and IκB-α with β-actin used as a control (**A**). Western blot densitometry analysis of phosphorylated NF-κB p65 (**B**) and phosphorylated IκB-α (**C**). Data are reported as means±SE. *P≤0.05 compared to complete Freund’s adjuvant (CFA)-induced group (ANOVA).

The effect of rhoifolin on the transcription factor NF-κB was tested on CFA-induced arthritic rats. Western blot analysis showed a significantly large increase in the levels of both NF-κB p65 and IκB-α in the synovial cavity of CFA rats (Figure 5). Treatment with 20 mg/kg rhoifolin caused a significant decrease in the levels of phosphorylated forms of NF-κB and IκB-α, however the decrease was not significant in the 10 mg/kg group.

### Effect of rhoifolin on rat knee histopathology

**Figure 6:**
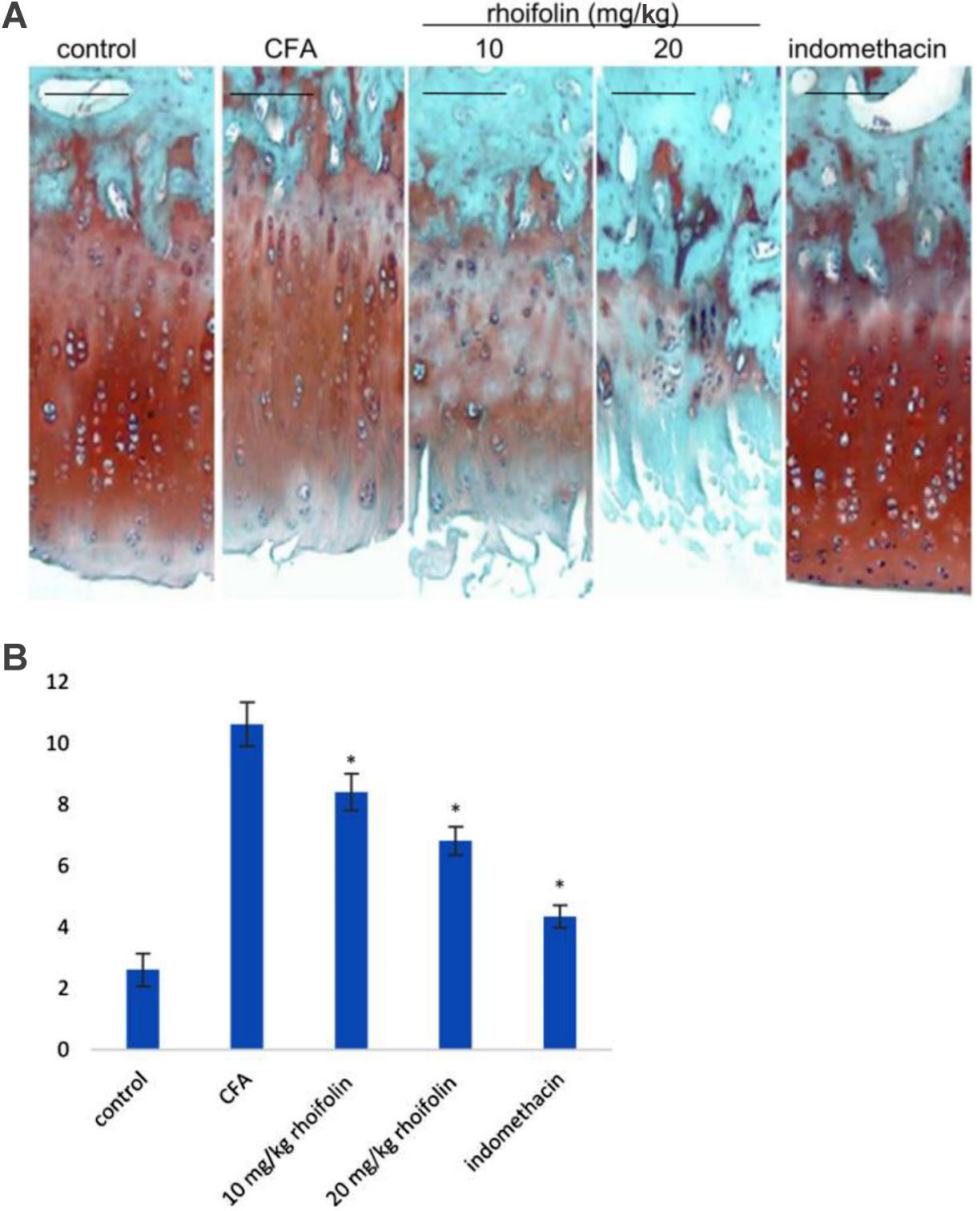
Histopathological analysis of the effect of rhoifolin treatment on complete Freund’s adjuvant (CFA)-induced arthritis in rat knee. **A**, Tissue sections of knee stained with hematoxylin and eosin and safranin O/Fast green. **B**, Morphological differences between the sections of different treatment groups scored following the Mankin scoring system (Supplementary Table S1). *P≤0.05 compared to CFA-induced group (ANOVA). Scale bar, 100 µM.

The histopathology of the healthy group was normal. However, the histopathology of the CFA-induced arthritis group showed extensive hypocellularity and deep fissures. The histopathology of the 10 mg/kg rhoifolin group showed a slight improvement over the CFA-induced arthritis group. The histopathology of 20 mg/kg rhoifolin and indomethacin groups showed significant improvement compared to the CFA group (Figure 6A). Mankin scores of the histopathological observations are plotted in Figure 6B, showing a significant improvement in the Mankin score of the rhoifolin treatment group compared to the CFA group.

## Discussion

Rheumatoid arthritis is an auto-immune disorder affecting joints and is associated with the inflammation of joints leading to severe pain. Almost 1% of the global population is reported to be affected by RA (10). At present, NSAIDs, methotrexate, and immunobiological agents are the only reliable options for the treatment of RA (14). Methotrexate is one of the oldest known immunosuppressants used for the treatment of RA. However, methotrexate therapy comes with several adverse effects such as gastrointestinal side effects and liver toxicity (28). Similarly, immunobiological agents such as rituximab, abatacept, and tocilizumab also have several adverse effects (29). Therefore, the development of safer effective therapeutic agents for the treatment of RA is warranted (30). In this study, we tested rhoifolin for acute toxicity as well as hepatic and kidney toxicity. Rhoifolin was found to be safe on these parameters.

Several flavonoids have been reported to possess anti-arthritic properties but very few have reached the clinical stage due to lack of understanding of the mechanism of their action (14). In this study, we attempted to identify the effect of anti-inflammatory plant flavonoid rhoifolin on CFA-induced RA rats. We also attempted to understand the possible mechanism of action of rhoifolin against RA. Adjuvant-induced arthritis has been shown to be the best strategy for the study of anti-arthritic drugs (31,32). The chronic polyarthritis induced by CFA closely resembles the symptoms of RA (33). Therefore, the CFA-induced arthritis model has been widely used for pre-clinical testing of several therapeutics for the treatment of RA. In this study, we report for the first time the effect rhoifolin on attenuating inflammation in RA by estimating edema, levels of cytokines TNF-α, IL-1β, and IL-6, and the transcription factor NF-κB. The anti-inflammatory action of rhoifolin on CFA-induced arthritis was also evaluated by histopathological analysis.

In this study, the first evidence indicating the anti-inflammatory potential of rhoifolin against CFA-induced RA was provided by the significant attenuation of edema in paw. A consistent decrease in the paw diameter in the rhoifolin-treated animals was observed over the experimental period. The decreased paw edema indicated humoral response inhibition by rhoifolin leading to decreased membrane permeability. The decrease in membrane permeability was probably mediated by the inhibition of pro-inflammatory cytokines by rhoifolin (34). This improvement in inflammation also had an effect on the overall health of the animals as the rhoifolin-treated animals showed an insignificant loss of weight compared to the healthy group. The improvement in these morphological parameters was corroborated by the histopathological analysis of the knee tissues. The histopathology of CFA-induced knee cartilage showed a significant improvement upon treatment with rhoifolin. We hypothesized that these changes occurred due to the attenuation of the pro-inflammatory signal.

The up-regulation of pro-inflammatory cytokines is a hallmark of RA inflammation. The effect of rhoifolin treatment on the expression of pro-inflammatory cytokines was observed to see if the attenuation of edema was a result of cytokine inhibition. The significant decrease in the levels of TNF-α, IL-1β, and IL-6 confirmed a positive effect of rhoifolin on alleviating inflammation in CFA-induced arthritis. In addition to inflammatory mediators, elevated oxidative stress has also been reported in RA (4,5). We used GSH, GPx, MDA, and SOD levels as markers for the estimation of oxidative stress in the articular chondrocytes. Rhoifolin showed a strong antioxidant response in CFA-induced arthritis, as evident from a significant decrease in these oxidative stress markers in the rhoifolin treatment groups compared to the non-treated group. Therefore, to elucidate the mechanism of the observed anti-arthritic action of rhoifolin, the levels of NF-κB were estimated in articular chondrocytes. Rhoifolin treatment showed a significant decrease in the NF-κB levels compared to the non-treated group. The transcription factor NF-κB is a key mediator of inflammatory responses (11). In addition to the expression of pro-inflammatory cytokines, it also helps in the amplification of the inflammatory signal on the account of a cytokine feedback loop. These results further strengthened our hypothesis that the anti-inflammatory and antioxidative properties of rhoifolin in the CFA-induced arthritis model are mediated by the NF-κB pathway.

In the present study, rhoifolin was found to inhibit the expression of pro-inflammatory cytokines and the NF-κB pathway in the knee tissue of CFA-induced arthritis in rats. The inhibition of cytokines and NF-κB was found to be downregulated at the gene expression and intracellular protein concentration levels. This reduced expression of pro-inflammatory cytokines may have caused the improvement in the gross morphology of articular cartilage. Rhoifolin also showed antioxidant properties as evident from the downregulation of oxidative stress markers. The study of rhoifolin toxicity on liver and kidney showed that rhoifolin had minimal adverse effects on these vital organs.

Therefore, the results of this study clearly showed that rhoifolin can be a potentially safe alternative to the extant therapies for the treatment of RA. Moreover, we propose that rhoifolin can potentially be used as a complementary or combination drug with the extant therapeutics. However, further studies are required to estimate the attenuation of pain parameters resulting from the attenuation of reduced inflammation by rhoifolin as shown in this study. Additional studies will be required to identify the underlying mechanism of rhoifolin action on RA such as the identification of its target molecules.

## Supplementary Material

Click here to view [pdf].
